# Bolus administration of remimazolam was superior to midazolam for deep sedation in elderly patients undergoing diagnostic bronchoscopy: A randomized, double-blind, controlled trial

**DOI:** 10.1097/MD.0000000000037215

**Published:** 2024-03-22

**Authors:** Qiuyue Wu, Rong Xu, Xuefei Zhou, Longfei Wang, Cheng Sheng, Miao Ding, Yunfei Cao

**Affiliations:** a School of Medicine, Ningbo University, Ningbo, China; b Department of Anesthesiology, Beilun District People’s Hospital of Ningbo, Ningbo, China; c Department of Anesthesiology, Qilu Hospital (Qingdao), Cheeloo College of Medicine, Shandong University, Qingdao, China.

**Keywords:** alfentanil, flexible bronchoscopy, midazolam, remimazolam, sedation

## Abstract

**Background:**

To date, there is no standardized practice for the use of pharmacological sedatives during flexible bronchoscopy, particularly for elderly patients. This exploratory study aimed to assess the efficacy and safety of remimazolam at a single induced dose for deep sedation in elderly patients undergoing diagnostic flexible bronchoscopy (DFB), and compare with midazolam, a commonly used sedative.

**Methods:**

A total of 100 elderly patients (age range 65–80 yr; American Society of Anesthesiologists Physical Status I–III) undergoing DFB were randomly allocated into 2 groups according to the sedatives used for induction: the remimazolam group and the midazolam group. Sedation induction was initiated by an intravenous bolus of remimazolam (0.135 mg/kg) or midazolam (0.045 mg/kg), respectively, both groups were combined with a high-dose of alfentanil (18 µg/kg), and supplemented with high-flow nasal cannula (HFNC) oxygen supply at a flow rate of 45 L/min. If the target depth of sedation was not achieved, propofol would be titrated as a rescue. The primary outcome was the success rate of sedation at a single induced dose to achieve target depth (Ramsay sedation score [RSS] = 4) during induction, intraoperative changes in vital signs, postoperative follow-up situation and incidence of post-bronchoscopy adverse events were evaluated as secondary outcomes.

**Results:**

The success rate of sedation in the remimazolam group was significantly higher than that in the midazolam group (65.2% vs 39.6%, *P* = .013), while the incidence of extra sleep within 6 hours after procedure was lower in the remimazolam group as compared to the midazolam group (10.9% vs 31.3%, *P* = .016). No statistically significant differences were observed between the 2 groups regarding hemodynamic fluctuations, incidence of hypoxemia, and cough response during the procedure, as well as postoperative recall, willingness to undergo reexamination, and other post-bronchoscopy adverse events.

**Conclusions:**

Bolus administration of remimazolam offers advantages over midazolam for deep sedation in elderly patients undergoing DFB, in terms of a higher success rate of sedation and a lower incidence of extra sleep within 6 hours after procedure, though the safety profiles of both groups were favorable.

## 1. Introduction

Flexible bronchoscopy (FB) is an essential, invasive procedure used in clinical practice for the diagnosis and treatment of bronchial, pulmonary, thoracic, and other diseases in clinical practice.^[1,2]^ Owing to concern of violent stimulation during FB procedure, sedation is recommended by most guidelines or expert consensus to be offered to all patients without contraindications.^[2,3]^ However, for elderly patients with fragile cardiopulmonary function, hypometabolism, and increased central sensitivity to anesthetic drugs, there are few preferred pharmacological agents for bronchoscopy sedation. Commonly used sedatives, such as propofol (associated with noticeable circulatory and respiratory depression), dexmedetomidine (with a prolonged induction time and susceptibility to hypotension and bradycardia), and midazolam (slow onset and long duration of action), have drawbacks that restrict their application in elderly patients.^[4,5]^ Fortunately, several emerging agents are being considered as acceptable alternatives, such as remimazolam, a new benzodiazepine for intravenous use in procedural sedation, has the rapid onset–offset properties comparable to propofol but with minor respiratory/circulatory depression.^[6,7]^ Some studies have confirmed that remimazolam could be safely and effectively used for bronchoscopy and had a faster onset of action and faster recovery of neuropsychiatric functions compared to midazolam.^[8,9]^ However, whether remimazolam may be the preferred pharmacological agents for bronchoscopy sedation in elderly patients has not been reported in the literature. The present study aimed to compare the efficacy and safety of remimazolam versus midazolam at a single induced dose for deep sedation in elderly patients undergoing diagnostic flexible bronchoscopy (DFB).

## 2. Patients and methods

### 2.1. Study design and patients

This was a randomized, double-blind, controlled trial. The protocol was approved by the institutional Ethics Committee of Beilun District People’s Hospital (Approval number: 2021-09[YS]) and registered to chictr.org (ChiCTR2100049052). The clinical trial was conducted in accordance with the Declaration of Helsinki between May 2022 and July 2022 at Beilun District People’s Hospital, an 840-bed tertiary care teaching hospital. Written informed consent was obtained from all participants before enrollment.

A total of 100 elderly patients (age range 65–80 yr; American Society of Anesthesiologists [ASA] physical status I–III) were recruited, and finally 94 were eligible and analyzed for this study. Indications for DFB included pneumonia (58.5%), bronchiectasis (20.2%), pulmonary shadow (6.4%), hemoptysis (6.4%), and miscellaneous (8.5%). The exclusion criteria were as follows: patients with measured peripheral capillary oxygen saturation (SpO_2_) <90% in room air; patients with a history of alcohol abuse or current use of any psychiatric medication; patients with severe cardiac disease, including aortic stenosis, mitral stenosis, haemodynamic instability caused by severe arrhythmia, and acute myocardial infarction or cardiac surgery within the last 6 months; patients with neurologic disorders (schizophrenia, Parkinson’s disease, or myasthenia gravis) or other conditions contributing to difficulty in assessing a conscious response; patients refusing to give informed consent.

Patients undergoing DFB were randomly allocated into either the remimazolam group or the midazolam group in a 1:1 ratio. A randomized allocation sequence was created by an independent research assistant who was not involved in the study using a random number sequence generated from a computer, and the study drug was prepared outside the bronchoscopy suite by a nurse uninvolved in the DFB procedure, based on the closed envelope method. The syringe of remimazolam or midazolam was labeled with the patient’s inclusion number with the same volume (dispensed with normal saline to 15 mL) and then passed to the anesthesiologist in charge of sedation. Patients, anesthesiologist in charge of sedation, as well as bronchoscopists were blinded to the sedation regimen. The demographic information, vital signs, and follow-up data of the patients were recorded on a paper case report form by an attending anesthesiologist.

### 2.2. Procedure and sedation

All patients were fasted for 8 hours for solids and for 2 hours for clear fluids without any premedication before the procedure. On the morning of their scheduled DFB, routine monitoring, including electrocardiogram, noninvasive blood pressure, respiratory rate, pulse oxygen saturation and end-tidal carbon dioxide, were performed upon the arrival of each patient in the operating room. For the purpose of topical anesthesia, patients all received 5 mL of 2% lidocaine induced by nebulized inhalation for 15 minutes before procedure, and 1 mL of 2% lidocaine solution were applied by nasal swap in each nose after the patients were lying on their dorsal position. All patients received humidified oxygen at a flow rate of 45 L/min and a concentration of 100% using a high-flow nasal cannula (HFNC) (AIRVO_2_, Fisher & Paykel, New Zealand) for 2 minutes before the commencement of sedation. Then, either a single dose of midazolam (0.045 mg/kg) (Nhwa Pharmaceutical Co., Ltd., Jiangsu, China, lot number: H19990027) or remimazolam (0.135 mg/kg) (Hengrui Pharmaceuticals Co., Ltd., Jiangsu, China, lot number: H20190034) was intravenously administered using a syringe pump at a speed of 20 mL/min (equivalent to 1200 mL/h). 1-minute later, a bolus of intravenous alfentanil (18 µg/kg) (Yichang Renfu Pharmaceuticals Co., Ltd., Yichang, China, lot number: H20190034) was administered using the same syringe pump technique. The target depth of sedation was set as a Ramsay sedation score (RSS) = 4 (Patient asleep, shows no response to verbal or tactile stimulus), if achieved, sedation was considered successful, otherwise propofol would be titrated as a rescue until the RSS score achieved 4. Then, DFB procedure was performed by the same experienced bronchoscopists (a chief physician in the department of respiratory at our hospital) using EVIS LUCERA BF-260 series bronchoscope (BF260, BF1T260, and BF-P260F; Olympus; Tokyo, Japan) via the nasal route, and 2% lidocaine was sprayed through the bronchoscope channel to enhance topical anesthesia with the “spray-as-you-go” technique over the vocal cords and the tracheobronchial tree. Various diagnostic procedures, including bronchial biopsy, bronchial brushing, and bronchoalveolar lavage, were performed based on individual clinical conditions. All DFB procedures were completed by 12:00 noon.

During DFB procedure, intraoperative (From the end of induction until the patients regained consciousness) hypertension/hypotension was defined as a systolic blood pressure more than 160 mm Hg/less than 90 mm Hg, and then urapidil/dopamine was given intravenously 1 mg each time until blood pressure stabilized. And episodes of hypoxemia, defined as an SpO_2_ <90% for >10 seconds, would be treated with Jaw thrust maneuver or temporary mask ventilation, and if hypoxemia remained uncorrected, laryngeal mask insertion or tracheal intubation would be determined by the anesthesiologist.

At the end of the bronchoscopy procedure, the specific antagonist of flumazenil (5 μg/kg) and naloxone (3 μg/kg) would be given immediately. Patients were transferred to the recovery room as they regained consciousness (RSS ≦ 2), and continuously monitored and received supplemental oxygen (4 L/min) through a conventional nasal catheter for 15 minutes. Subsequently, they were transferred to the inpatient ward and with a 3 hours constant monitoring. Postoperative follow-up was performed 6 hours after the procedure to avoid any judgement alteration due to residual effects of the drugs.

### 2.3. Data collection and measured outcomes

The primary outcome assessed was the success rate of sedation at a single induced dose, defined as achieving the target depth of sedation (RSS = 4) with a single induced dose of sedatives and no need for a rescue sedative medication (propofol). The secondary outcomes included intraoperative changes in vital signs, such as hemodynamic stability and the use of vasoactive drugs, the incidence of hypoxemia and the minimum SpO_2_ value, cough severity evaluated by visual analogue scale (VAS) with a score from 0 to 10 indicating no cough to severe cough, induction time was defined as the initiation of bolus sedatives to the beginning of the FB procedure, while recovery time was defined as the end of the procedure and administration of antagonists (flumazenil and naloxone) until the patient’s RSS score ≦ 2. Postoperative follow-up situations were also used as secondary outcomes, such as incidence of postoperative extra sleep within 6 hours postoperatively, postoperative recall and willingness to undergo reexamination, and post-bronchoscopy adverse events, such as nausea, vomiting, fatigue, and dizziness.

### 2.4. Statistical analysis

Statistical analyses were conducted using SPSS Statistics Version 25.0 (IBM Co.). The data are presented as mean (standard deviation) for continuous variables and as the number of patients (%) for categorical variables. Continuous data were analyzed using independent sample *t* test, while categorical data were analyzed using the chi-squared test or Fisher’s exact test, as appropriate. A significance level of *P* < .05 was used to determine statistical significance.

## 3. Results

### 3.1. Demographic characteristics

**Figure 1.**
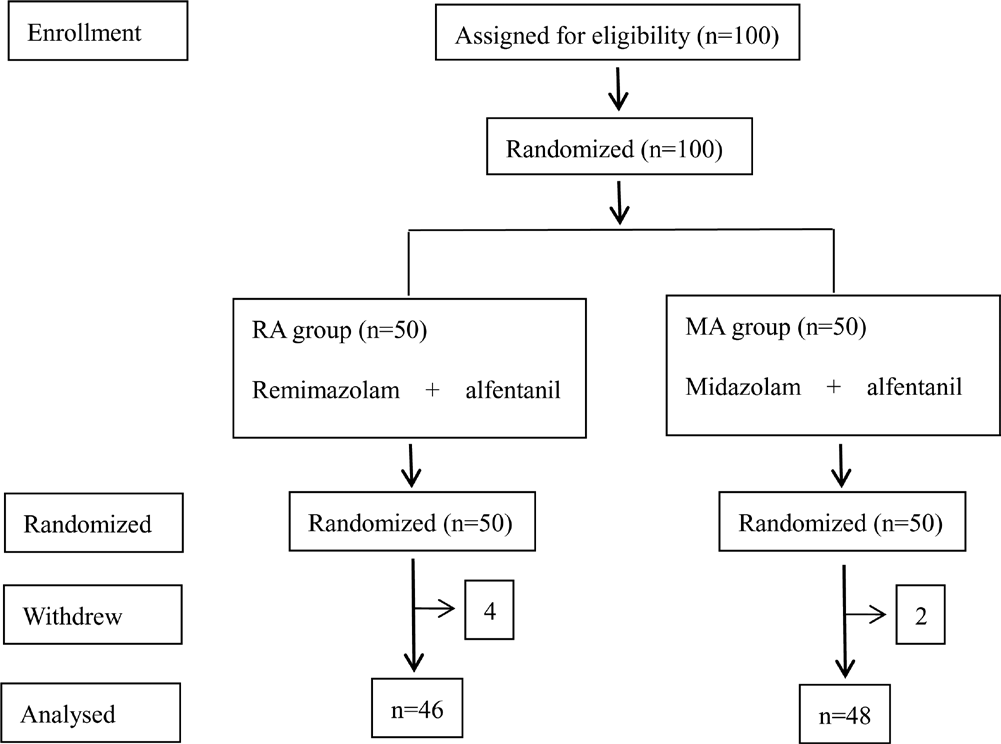
The flow diagram of the study.

Of the 100 patients randomized into the study, 6 withdrew before completion, as described in Figure 1. Four patients in the remimazolam group withdrew from the trial, one of them was converted to transoral FB procedure due to bilateral nasal meatus stenosis, 2 patients were converted to mass resection due to endobronchial masses found during FB procedure, and 1 patient was converted to ultrasound-guided transbronchial needle biopsy due to intrabronchial abnormalities found during FB procedure. While in the midzolam group, only 2 patients withdrew from the trial, 1 was converted to mass resection due to endobronchial masses and 1 to ultrasound-guided transbronchial needle biopsy due to intrabronchial abnormalities found during FB procedure.

There were no obvious differences in baseline characteristics (age, sex, weight, body mass index (BMI), and ASA physical status) between the 2 groups (*P* > .05), as shown in Table 1. The primary indications for DFB were pneumonia, followed by bronchiectasis, lung shadow and hemoptysis. Diagnostic procedures included bronchoalveolar lavage, bronchial brushing and bronchial biopsy. The indications and procedures were uniformly distributed in both groups (*P* > .05), as shown in Table 1.

**Table 1:** Characteristics of the 2 groups for eligible patients.

	RA group (n = 46)	MA group (n = 48)	*P* value
Gender male/female	26/20	29/19	.702
ASA classification I/II/III	6/33/7	7/32/9	.989
Age (yr)	70.37 ± 4.07	69.21 ± 3.59	.145
Weight (kg)	59.33 ± 11.91	60.67 ± 10.53	.564
BMI (kg/m^2^)	22.09 ± 3.65	22.19 ± 3.06	.885
Indications for DFB			
Pneumonia	27 (58.7%)	28 (58.3%)	.846
Bronchiectasis	9 (19.6%)	10 (20.8%)	
Lung shadow	4 (8.7%)	2 (4.2%)	
Hemoptysis	2 (4.3%)	4 (8.3%)	
Miscellaneous	4 (8.7%)	4 (8.3%)	
Procedures for DFB			.155
BAL	2 (4.3%%)	7 (14.6%)	
BAL + BBr	41 (89.1%)	36 (75%)	
BAL + BBr + BBi	3 (6.5%)	5 (10.4%)	

ASA = American Society of Anesthesiologists, BAL = bronchoalveolar lavage, BBi = Bronchial biopsy, BBr = Bronchial brushing, BMI = body mass index, DFB = diagnostic flexible bronchoscopy.

### 3.2. Intraoperative clinical data and outcome measures

All eligible patients in both groups underwent diagnostic evaluation of the tracheobronchial tree successfully and woke up within 7 minutes after bronchoscopy. The mean induction time and total inspection time of bronchoscopy in group RA were 3.9 ± 1.2 minutes and 5.7 ± 2.2 minutes respectively, compared with 4.2 ± 1.4 minutes and 5.6 ± 2.3 minutes in group MA, and no significant differences were found between the 2 groups (*P* > .05). The success rate of sedation with bolus administration of remimazolam was significantly higher than that with midazolam (65.2% vs 39.6%, *P* = .013). There were no statistically significant differences between the 2 groups in terms of circulatory fluctuations (proportion of patients using urapidil or dopamine), incidence of hypoxemia (8.7% vs 8.3%), minimum SpO_2_ value, cough VAS score (1.4 ± 1.3 vs 1.2 ± 1.1), and awake time between the 2 groups, as shown in Table 2.

**Table 2:** Clinical data of patients during DFB procedure.

	RA group (n = 46)	MA group (n = 48)	*P* value
Primary outcome			
The success rate of sedation	30 (65.2%)	19 (39.6%)	.013
Secondary outcomes			
The dosage of remimazolam (mg)	NA	8.0 ± 1.6	NA
The dosage of midazolam (mg)	2.7 ± 0.5	NA	NA
The dosage of alfentanil (µg)	1062.5 ± 212.8	1084.4 ± 182.2	.593
Patients receiving			
Propofol	16 (34.8%)	29 (60.4%)	.013
Urapidil	3 (6.5%)	2 (4.2%)	.961
Dopamine	5 (10.9%)	1 (2.1%)	.187
Induction time (min)	3.9 ± 1.2	4.2 ± 1.4	.221
Total inspection time (min)	5.7 ± 2.2	5.6 ± 2.3	.775
Cough VAS score	1.4 ± 1.3	1.2 ± 1.1	.517
Minimum SpO_2_ (%)	97.5 ± 5.5	97.5 ± 6.7	.960
Patients with			
Hypoxemia	4 (8.7%)	4 (8.3%)	1.000
Anti-hypoxic interventions			
Jaw thrust maneuver	2 (4.3%)	2 (4.2%)	1.000
Temporary mask ventilation	2 (4.3%)	2 (4.2%)	
Intubation or LMA insertion	0	0	
Patients with			
Awake time (min) 0/1/2/3/4/≧5	32/4/2/6/1/1	28/4/8/4/4/0	.168

LMA = laryngeal mask airway, NA = not available, SpO_2_ = saturation of peripheral oxygen, VAS = visual analogue scale.

### 3.3. Follow-up (6 h after the procedure) and adverse events

Within 6 hours postoperatively, the incidence of extra sleep in remimazolam group was lower than that in midazolam group (10.9% vs 31.3%, *P* = .016), but no statistically significant differences were observed between the groups regarding postoperative recall and willingness to undergo reexamination, as well as other post-bronchoscopy adverse events, including nausea, vomiting, fatigue, and dizziness (*P* > .05, see Table 3).

**Table 3:** Postoperative follow-up and adverse events.

	RA group (n = 46)	MA group (n = 48)	*P* value
Patients with:			
Postoperative recall	0 (0%)	1 (2.1%)	1.000
Willingness to return	44 (95.7%)	45 (93.8%)	1.000
Postoperative extra sleep	5 (10.9%)	15 (31.3%)	.016
Nausea	3 (6.5%)	2 (4.2%)	.961
Vomiting	1 (2.2%)	0 (0%)	.983
Fatigue	7 (15.2%)	5 (10.4%)	.486
Dizziness	5 (10.9%)	5 (10.4%)	1.000

## 4. Discussion

Our prospective randomized controlled study showed for the first time that bolus administration of remimazolam was superior to midazolam for deep sedation in elderly patients undergoing DFB, mainly in terms of higher success rate of sedation at a single induced dose to achieve target level of deep sedation, and lower incidence of extra sleep within 6 hours postoperatively, though both sedation regimens are relatively safe and effective when combined with high doses of alfentanil and supplemented with remedial sedatives and high-flow humidified oxygen. Considering single intravenous bolus induction is usually required for its convenience in clinical practice, our findings may provide valuable insights into the selection of sedative agents or regimens for DFB in elderly patients.

Regarding the comparison of sedative efficacy of these 2 benzodiazepines, many literatures believed that remimazolam was superior to midazolam. Studies by Keam SJ showed that the onset of remimazolam was significantly faster than midazolam, and non-inferior to propofol.^[10]^ The comparative study of adult bronchoscopy under moderate sedation conducted by Pastis NJ also confirmed that, the sedation success rate of remimazolam (80.6%) was significantly higher than that of midazolam (32.9%).^[11]^ However, there have been no comparative studies of these 2 benzodiazepine**s** for sedation in elderly patients undergoing bronchoscopy. Our study showed that, as compared to midazolam (0.045 mg/kg), intravenous bolus injection of remimazolam (0.135 mg/kg) demonstrated a higher success rate in achieving target level of deep sedation, the results were consistent with the previously published studies in adults.^[11]^ However, it should be emphasized that the doses of the 2 benzodiazepine sedatives involved in this study were not equipotent. To date, the equipotent dose of midazolam and remimazolam is not known, especially in elderly patients and in combination with afentanil. The doses of midazolam (0.045 mg/kg) and remimazolam (0.135 mg/kg) used in this study were determined based on guidelines and expert consensus, as well as our previous clinical experience.^[2,4,12]^ The per capita dose of midazolam was 2.7 mg in our study, which has exceeded the recommended dose for elderly patients in the British Thoracic Society Guidelines.^[13]^ As for remimazolam, the optimal doses required for the induction of general anesthesia or procedural sedation for endoscopy in elderly patients remain unclear. In Chinese patients, the ED 95 of remimazolam tosilate for anesthesia induction was 0.118 mg/kg (95% CI 0.103–0.649) and 0.090 mg/kg (95% CI 0.075–0.199) in elderly patients aged 60 to 69 and 70 to 85 years, respectively.^[14]^ Obviously, the dose of remimazolam (0.135 mg/kg) adopted for bolus administration in our study were higher than these ED 95 values, and even in combination with a fixed high dose of afentanil, 34.8% of elderly patients still failed to achieve the target level of deep sedation. Therefore, the optimal doses required for DFB sedation in elderly patients need to be further observed and clarified. But it’s important to note that, although our findings showed that both sedation regimens of remimazolam and midazolam were relatively safe, it is clear that further increases in the doses of these 2 benzodiazepines could easily lead to a dramatic increase in the risk of hypoxemia and post-bronchoscopy adverse events.

Most guidelines and expert consensus recommend mild to moderate sedation (RSS between 2 and 3 points) for bronchoscopy.^[2,4,13,15]^ The rationale behind this recommendation is the concern that deep sedation or oversedation may lead to significant respiratory and circulatory depression. Moreover, desaturation risk with moderate sedation is particularly prone to occur in elderly patients and those with an ASA III score.^[16,17]^ However, in many cases, mild to moderate sedation is hard to effectively inhibit the intense airway irritation and severe cough caused by bronchoscopic procedure, resulting in significant intraoperative circulatory fluctuations and cardiovascular hazards, which cannot be ignored especially for elderly patients with fragile cardiovascular function. In this context, the maintenance of intraoperative circulatory stability in the elderly patients is the focus of perioperative sedation or anesthesia.^[18,19]^ Our results showed that the circulation fluctuation and cough VAS scores in both groups were relatively low, and severe cough with a VAS score exceeding 5 was quite rare, which could be attributed to the co-administration of high doses of alfentanil and deep sedation level. It is widely acknowledged that the typical sedation regimens for FB is difficult to solve cough and stress problems in the absence of opioids.^[3,13,15]^ As a short-acting opioid with mild respiratory depression, alfentanil possesses notable anti-stress and anti-coughing effects, but only at high doses can the desired effect be achieved, which has been confirmed in our previous research and other literatures.^[12,20,21]^ In this study, the combined application of high doses of alfentanil proved effective in achieving deep sedation and contributed to ideal anti-stress effect and hemodynamic stabilization. Notably, the numbers of patients required vasoactive drugs (urapidil or dopamine) administered intraoperatively due to hypertension or hypotension were very few. Thus, it was evident that for elderly patients with fragile cardiopulmonary function, the application of hgh doses of afentanil was safe and feasible. Additionally, we advocate for propofol as a preferred rescue choice when the target sedation depth is not achieved after bolus administration of sedatives and alfentanil, for low doses of propofol supplemented will not cause significant respiratory and circulatory depression,^[22]^ and helps to avoid prolonged postoperative lethargy or delayed cognitive recovery due to excessive doses of remimazolam or midazolam.^[23,24]^

In this study, deep sedation with non-intubation and preserved spontaneous breathing was adopted for elderly bronchoscopy patients, and no similar literature has been reported so far. Though at the same level of deep sedation, patients in both groups showed comparable hemodynamic stability, minimal hypoxic incidence, as well as satisfactory bronchoscopy conditions, and high willingness to undergo reexamination. However, the major risks of respiratory depression and hypoxemia associated with deep sedation should be emphasized. In order to effectively prevent the hypoxic risk, a new oxygen supply technique, namely HFNC, was also used in this study, which can significantly improve the safety of deep sedation and further improve the conditions of bronchoscopy manipulation.^[24,25]^ In our study, HFNC set at a flow rate of 45 L/min substantially reduced the incidence of hypoxemia to less than 10% in both 2 groups under deep sedation, and in all cases in which hypoxia occurred, normal oxygenation was rapidly restored only by jaw support or temporary mask-assisted ventilation, and none required laryngeal mask insertion or endotracheal intubation. The anti-hypoxia effect of HFNC has been confirmed in many studies, a study from Spence EA found that the effectiveness of HFNC in improving oxygenation and prolongating the duration of safe apnea during general anesthesia induction.^[26]^ HFNC serves multifaceted functions, such as reducing the anatomic dead space of the nasopharynx, exerting a positive end-expiratory pressure effect, augmenting end-expiratory lung volume, and ensuring effective alveolar ventilation to prevent atelectasis.^[24–26]^ Moreover, HFNC also helps to avoid insufficient ventilation and partial airway obstruction caused by sedation with high doses of opioids.^[27]^ These effects emphasize the pivotal role of HFNC in enhancing the safety and efficacy of bronchoscopy under deep sedation.

Our results also confirmed that the incidence of extra sleep after procedure was significantly reduced in the remimazolam group than that in the midazolam group, this could be attributed to the prolonged sedative effect of midazolam and its active metabolites due to its organ dependency and considerably lower drug clearance rate, particularly in elderly patients. Williams’ studies confirmed that the average serum benzodiazepine concentration 2 hours after sedation was approximately 2-fold higher in patients aged ≥70 years than in those aged <40 years, and for this reason, elderly patients were likely to require lower doses of sedatives for bronchoscopy.^[28]^ Considering the short duration and intense irritation of bronchoscopy procedure, agents such as remimazolam, that provides an appropriate depth of sedation with a rapid onset and recovery, are preferrable. It is now clear that remimazolam is rapidly metabolized to a pharmacologically inactive metabolite by carboxylesterase-1A, independent of the cytochrome P450 enzyme, which makes the recovery from remimazolam sedation faster and more predictable than midazolam.^[29,30]^ Our study confirmed that both sedative drugs could be safely used and meet the needs of DFB sedation by bolus administration, and only one case had intraoperative painful recall found in postoperative follow-up, which may be related to the deep sedation level adopted in this study. Given the convenience of single intravenous bolus induction for procedural sedation, it is believable that the combined sedation regimen of remimazolam and alfentanil with bolus administration is feasible and superior in elderly patients.

This study presents several limitations. First, while our data analysis indicated the superiority of bolus remimazolam injection over midazolam, larger sample sizes, multicenter studies, as well as well-designed randomized controlled clinical trials are needed to confirm and support these findings. Second, patients with respiratory failure were excluded from the study. Therefore, the findings of this study may primarily pertain to patients with near normal respiratory function and not requiring pre-bronchoscopy oxygen supplementation. Third, the induction dose of midazolam used in this study should not be further increased, but whether the induction dose of remimazolam can be increased to improve the success rate of inducing sedation without causing other adverse reactions such as hypoxemia, delayed recovery and postoperative extra sleep, needs further observation and study. And last, both benzodiazepines and alfentanil can potentially lead to postoperative delirium or cognitive impairment.^[31,32]^ Although this study did not observe significant postoperative cognitive changes or postoperative delirium, this possibility cannot be entirely ruled out and warrants further observation.

## 5. Conclusion

With the assistance of high-efficiency HFNC for oxygen supply, both deep sedation regimens in this randomized trial can be safely used for DFB procedure in elderly patients. However, the bolus administration of remimazolam combined with alfentanil proves to be superior to midazolam-alfentanil regimen in terms of higher success rate of sedation at a single induced dose and lower incidence of extra sleep within 6 hours postoperatively. Thus, the combination of remimazolam and alfentanil emerges as a more favorable clinical choice.

## Author contributions

**Conceptualization:** Qiuyue Wu, Yunfei Cao.

**Data curation:** Rong Xu, Xuefei Zhou, Longfei Wang, Cheng Sheng, Miao Ding.

**Formal analysis:** Xuefei Zhou, Longfei Wang, Cheng Sheng.

**Funding acquisition:** Qiuyue Wu, Yunfei Cao.

**Investigation:** Qiuyue Wu, Rong Xu, Xuefei Zhou, Longfei Wang, Miao Ding.

**Methodology:** Rong Xu, Xuefei Zhou, Yunfei Cao.

**Project administration:** Yunfei Cao.

**Supervision:** Yunfei Cao.

**Writing – original draft:** Qiuyue Wu, Rong Xu.

**Writing – review & editing:** Yunfei Cao.

## References

[1] Gaisl T Bratton DJ Heuss LT. Sedation during bronchoscopy: data from a nationwide sedation and monitoring survey. BMC Pulm Med. 2016;16:113. 27495824 10.1186/s12890-016-0275-4 PMC4974777

[2] Wahidi MM Jain P Jantz M. American College of Chest Physicians consensus statement on the use of topical anesthesia, analgesia, and sedation during flexible bronchoscopy in adult patients. Chest. 2011;140:1342–50. 22045879 10.1378/chest.10-3361

[3] McCambridge AJ Boesch RP Mullon JJ. Sedation in Bronchoscopy: a review. Clin Chest Med. 2018;39:65–77. 29433726 10.1016/j.ccm.2017.09.004

[4] Mohan A Madan K Hadda V. Jayachandra. Guidelines for diagnostic flexible bronchoscopy in adults: Joint Indian Chest Society/National College of chest physicians (I)/Indian association for bronchology recommendations. Lung India. 2019;36(Suppl):S37–89. 32445309 10.4103/lungindia.lungindia_108_19 PMC6681731

[5] Zhang Q Zhou J He Q. Dexmedetomidine combined with midazolam infusion guided by bispectral index during bronchoscopy. Clin Respir J. 2021;15:929–36. 33934514 10.1111/crj.13383

[6] Lee TY Kim MA Eom DW. Comparison of remimazolam-remifentanil and propofol-remifentanil during laparoscopic cholecystectomy. Anesth Pain Med (Seoul). 2023;18:252–9. 37468208 10.17085/apm.22252 PMC10410549

[7] Choi JY Lee HS Kim JY. Comparison of remimazolam-based and propofol-based total intravenous anesthesia on postoperative quality of recovery: a randomized non-inferiority trial. J Clin Anesth. 2022;82:110955. 36029704 10.1016/j.jclinane.2022.110955

[8] Kim SH Cho JY Kim M. Safety and efficacy of remimazolam compared with midazolam during bronchoscopy: a single-center, randomized controlled study. Sci Rep. 2023;13:20498. 37993525 10.1038/s41598-023-47271-w PMC10665376

[9] Dao VA Schippers F Stöhr T. Efficacy of remimazolam versus midazolam for procedural sedation: post hoc integrated analyses of three phase 3 clinical trials. Endosc Int Open. 2022;10:E378–85. 35433203 10.1055/a-1743-1936 PMC9010109

[10] Kim SH Fechner J. Remimazolam-current knowledge on a new intravenous benzodiazepine anesthetic agent. Korean J Anesthesiol. 2022;75:307–15. 35585830 10.4097/kja.22297 PMC9346281

[11] Pastis NJ Yarmus LB Schippers F. PAION Investigators. Safety and efficacy of remimazolam compared with placebo and midazolam for moderate sedation during bronchoscopy. Chest. 2019;155:137–46. 30292760 10.1016/j.chest.2018.09.015

[12] Wang L Wu Q Wang M. The safety and efficacy of alfentanil combined with midazolam in fiberoptic bronchoscopy sedation: a randomized, double-blind, controlled trial. Front Pharmacol. 2022;13:1036840. 36339547 10.3389/fphar.2022.1036840 PMC9634630

[13] Du Rand IA Barber PV Goldring J. British Thoracic Society Interventional Bronchoscopy Guideline Group. British Thoracic Society guideline for advanced diagnostic and therapeutic flexible bronchoscopy in adults. Thorax. 2011;66(Suppl 3):iii1–21. 21987439 10.1136/thoraxjnl-2011-200713

[14] Liu M Sun Y Zhou L. The median effective dose and bispectral index of remimazolam tosilate for anesthesia induction in elderly patients: an up-and-down sequential allocation trial. Clin Interv Aging. 2022;17:837–43. 35620021 10.2147/CIA.S364222 PMC9129099

[15] Minami D Takigawa N. Safe sedation during diagnostic and therapeutic flexible bronchoscopy in Japan: a review of the literature. Respir Investig. 2023;61:52–7. 10.1016/j.resinv.2022.09.003 36220691

[16] McCambridge AJ Boesch RP Mullon JJ. Sedation in bronchoscopy: a review. Clin Chest Med. 2018;39:65–77. 29433726 10.1016/j.ccm.2017.09.004

[17] Rex DK Bhandari R Lorch DG. Safety and efficacy of remimazolam in high-risk colonoscopy: a randomized trial. Dig Liver Dis. 2021;53:94–101. 33243567 10.1016/j.dld.2020.10.039

[18] Hinkelbein J Schmitz J Lamperti M. Procedural sedation outside the operating room. Curr Opin Anaesthesiol. 2020;33:533–8. 32628400 10.1097/ACO.0000000000000885

[19] Chae D Kim HC Song Y. Pharmacodynamic analysis of intravenous bolus remimazolam for loss of consciousness in patients undergoing general anaesthesia: a randomised, prospective, double-blind study. Br J Anaesth. 2022;129:49–57. 35562226 10.1016/j.bja.2022.02.040

[20] Chen N Wang X Chen L. Estimation of the median effective dose and the 95% effective dose of alfentanil required to inhibit the bronchoscopy reaction during painless bronchoscopy with i-gel supraglottic airway device: an up-and-down sequential allocation trial. J Thorac Dis. 2022;14:1537–43. 35693612 10.21037/jtd-22-412 PMC9186247

[21] Wang L Wu Q Wang M. Gender differences in the effective dose of alfentanil in painless bronchoscopy. J Thorac Dis. 2023;15:216–8. 36794143 10.21037/jtd-22-1460 PMC9922608

[22] Li YP Zhou Y. Differential dosing of oxycodone in combination with propofol in diagnostic painless gastroscopy in elderly patients: A prospective randomized controlled trial. Medicine (Baltimore). 2022;101:e32427. 36595823 10.1097/MD.0000000000032427 PMC9794329

[23] Sun H Wang T Xu ZX. Effective dose and adverse reactions analysis of remimazolam for sedation in elderly patients undergoing gastroscopy. Zhonghua Yi Xue Za Zhi. 2022;102:332–5. 35092973 10.3760/cma.j.cn112137-20211111-02509

[24] Longhini F Pelaia C Garofalo E. High-flow nasal cannula oxygen therapy for outpatients undergoing flexible bronchoscopy: a randomised controlled trial. Thorax. 2022;77:58–64. 33927023 10.1136/thoraxjnl-2021-217116

[25] Tao Y Sun M Miao M. High flow nasal cannula for patients undergoing bronchoscopy and gastrointestinal endoscopy: a systematic review and meta-analysis. Front Surg. 2022;9:949614. 36046260 10.3389/fsurg.2022.949614 PMC9420969

[26] Spence EA Rajaleelan W Wong J. The effectiveness of high-flow nasal oxygen during the intraoperative period: a systematic review and meta-analysis. Anesth Analg. 2020;131:1102–10. 32925331 10.1213/ANE.0000000000005073

[27] Pelaia C Bruni A Garofalo E. Oxygenation strategies during flexible bronchoscopy: a review of the literature. Respir Res. 2021;22:253. 34563179 10.1186/s12931-021-01846-1 PMC8464093

[28] Williams TJ. Sedation in fibreoptic bronchoscopy. Doses that produce amnesia should be given. BMJ. 1995;310:872. 10.1136/bmj.310.6983.872a PMC2549249 7711644

[29] Van-Anh D Frank S Thomas S. Efficacy of remimazolam versus midazolam for procedural sedation: post hoc integrated analyses of three phase 3 clinical trials. Endosc Int Open. 2022;10:E378–85. 35433203 10.1055/a-1743-1936 PMC9010109

[30] Lee A Shirley M. Remimazolam: a review in procedural sedation. Drugs. 2021;81:1193–201. 34196946 10.1007/s40265-021-01544-8

[31] Duprey MS Devlin JW Griffith JL. Association between perioperative medication use and postoperative delirium and cognition in older adults undergoing elective noncardiac surgery. Anesth Analg. 2022;134:1154–63. 35202006 10.1213/ANE.0000000000005959 PMC9124692

[32] Guenther U Riedel L Radtke FM. Patients prone for postoperative delirium: preoperative assessment, perioperative prophylaxis, postoperative treatment. Curr Opin Anaesthesiol. 2016;29:384–90. 26905874 10.1097/ACO.0000000000000327

